# Semaphorin 3A Inhibits Nerve Regeneration During Early Stage after Inferior Alveolar Nerve Transection

**DOI:** 10.1038/s41598-018-37819-6

**Published:** 2019-03-12

**Authors:** Hiroko Kanemaru, Yurie Yamada, Atsushi Ohazama, Takeyasu Maeda, Kenji Seo

**Affiliations:** 1 Division of Dental Anesthesiology, Niigata University Graduate School of Medical and Dental Sciences, Niigata, Japan; 2 Center for Advanced Oral Sciences, Niigata University Graduate School of Medical and Dental Sciences, Niigata, Japan; 3 Division of Oral Anatomy, Niigata University Graduate School of Medical and Dental Sciences, Niigata, Japan; 4 Faculty of Dentistry, University of Airlangga, Surabaya, Indonesia

## Abstract

Neuroma formation at sites of injury can impair peripheral nerve regeneration. Although the involvement of semaphorin 3A has been suggested in neuroma formation, this detailed process after injury is not fully understood. This study was therefore undertaken to examine the effects of semaphorin 3A on peripheral nerve regeneration during the early stage after injury. Immunohistochemistry for semaphorin 3A and PGP9.5, a general neuronal marker, was carried out for clarify chronological changes in their expressions after transection of the mouse inferior alveolar nerve thorough postoperative days 1 to 7. At postoperative day 1, the proximal stump of the damaged IAN exhibited semaphorin 3A, while the distal stump lacked any immunoreactivity. From this day on, its expression lessened, ultimately disappearing completely in all regions of the transected inferior alveolar nerve. A local administration of an antibody to semaphorin 3A into the nerve transection site at postoperative day 3 inhibited axon sprouting at the injury site. This antibody injection increased the number of trigeminal ganglion neurons labeled with DiI (paired t-test, p < 0.05). Immunoreactivity of the semaphorin 3A receptor, neuropilin-1, was also detected at the proximal stump at postoperative day 1. These results suggest that nerve injury initiates semaphorin 3A production in ganglion neurons, which is then delivered through the nerve fibers to the proximal end, thereby contributes to the inhibition of axonal sprouting from the proximal region of injured nerves in the distal direction. To our knowledge, this is the first report to reveal the involvement of Sema3A in the nerve regeneration process at its early stage.

## Introduction

Peripheral nerves have the potential for axonal regeneration upon injury or transection. Because central nervous system injuries such as stroke or spinal cord injury are difficult to heal, neural impairment is long lasting or permanent. This is because nerve injury forms a glial scar that disturbs axonal regrowth^1^. Furthermore, fibroblasts within the scar have the ability to produce the repulsive axon guidance molecule semaphorin 3A (Sema3A)^2^ which functions as an axonal guidance cue in embryos to effect the normal innervation of peripheral nerves^3^. It binds its specific receptor, neuropilin-1, to the surface of neurons to induce the inhibition of neurite regrowth, collapse of the growth cone, and axoplasmic transport^4^. However, in the peripheral nerve of mature humans or animals, nerve injury sometimes generates traumatic neuroma, inducing an intractable pain sensation^5^.

Dental treatments sometimes cause sensory impairment, especially in the region innervated by the third division of the trigeminal nerve, thereby leading to vulnerability to sensory deficit hypoesthesia in the orofacial region. We also reported neuroma formation in some patients with inferior alveolar nerve (IAN) injury subsequent to wisdom tooth extraction or oral implant surgery^6^. It is thus important to understand the peripheral nerve regeneration process and mechanism in order to develop new clinical treatment in dentistry. Previous studies have detected Sema3A in neuroma generated after brachial plexus surgery, suggesting the involvement of this molecule in neuroma formation^7^. Therefore, it is hypothesized that local control of Sema3A is a target for adequate peripheral nerve regeneration after injury. The present study was undertaken to clarify the possible involvement of Sema3A in peripheral nerve regeneration in a mouse experimental model^8–10^ at early stage following transection of the IAN. It shall focus on; (1) changes in expression pattern of Sema3A in the injured IAN and trigeminal ganglion neurons and (2) the effects of the anti-Sema3A antibody on the IAN regeneration.

## Results

### Early expression of Sema3A after IAN transection

**Figure 1:**
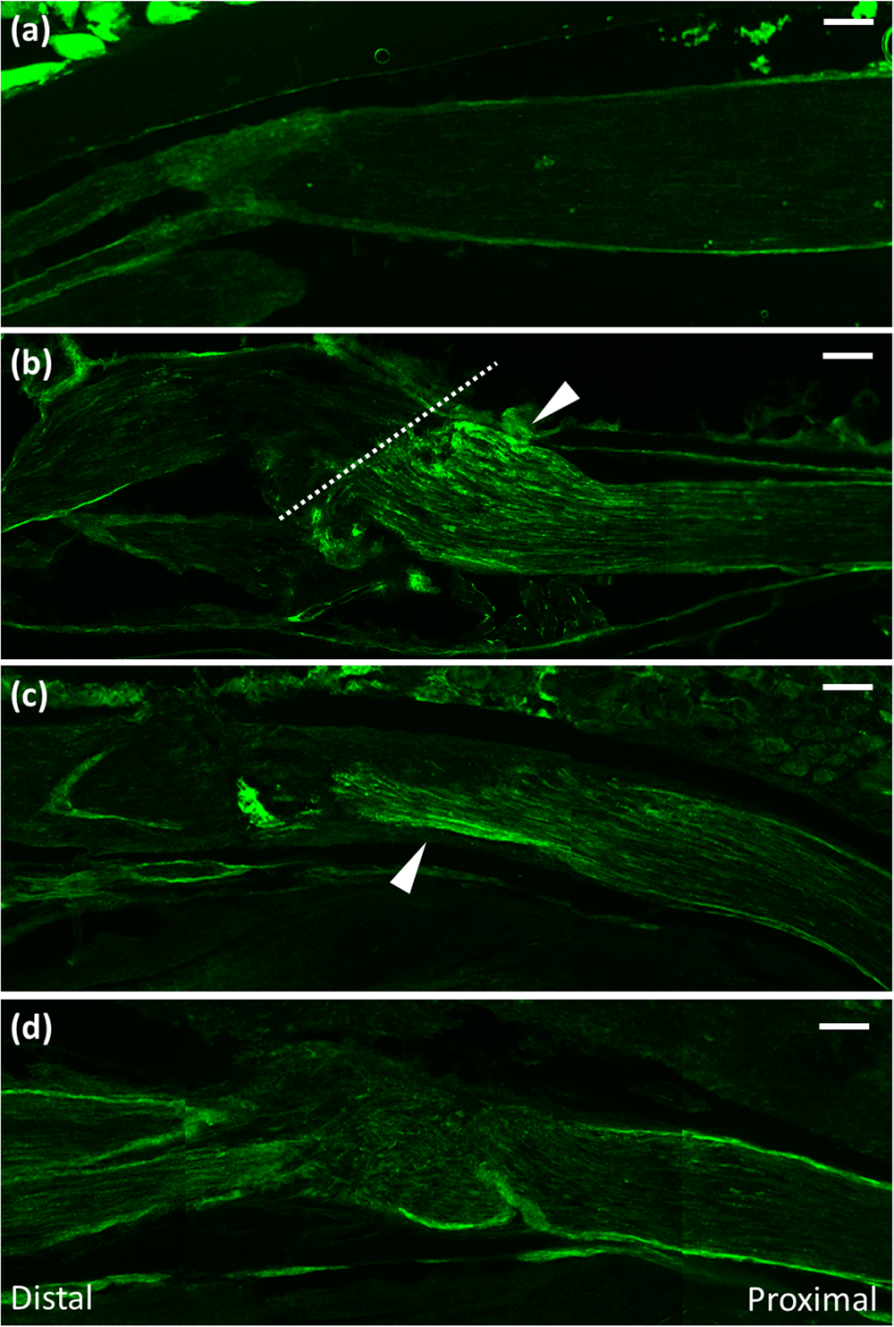
Expression of Sema3A during IAN regeneration. Sagittal sections exhibiting immunohistochemistry of Sema3A (**a**) before transection of IAN and at postoperative (PO) day 1(**b**), 3 (**c**), and 7 (**d**). A dotted line in (**b**) indicates the transection site. The expression of Sema3A is recognizable in the proximal nerve end of IAN at PO day 1 (arrowhead), and was reduced at PO day 3. At PO day 7, no Sema3A immunoreactivity exists in IAN. Scale bar = 100 µm. Left or right side in each picture indicates the distal or proximal direction of IAN, respectively.

**Figure 2:**
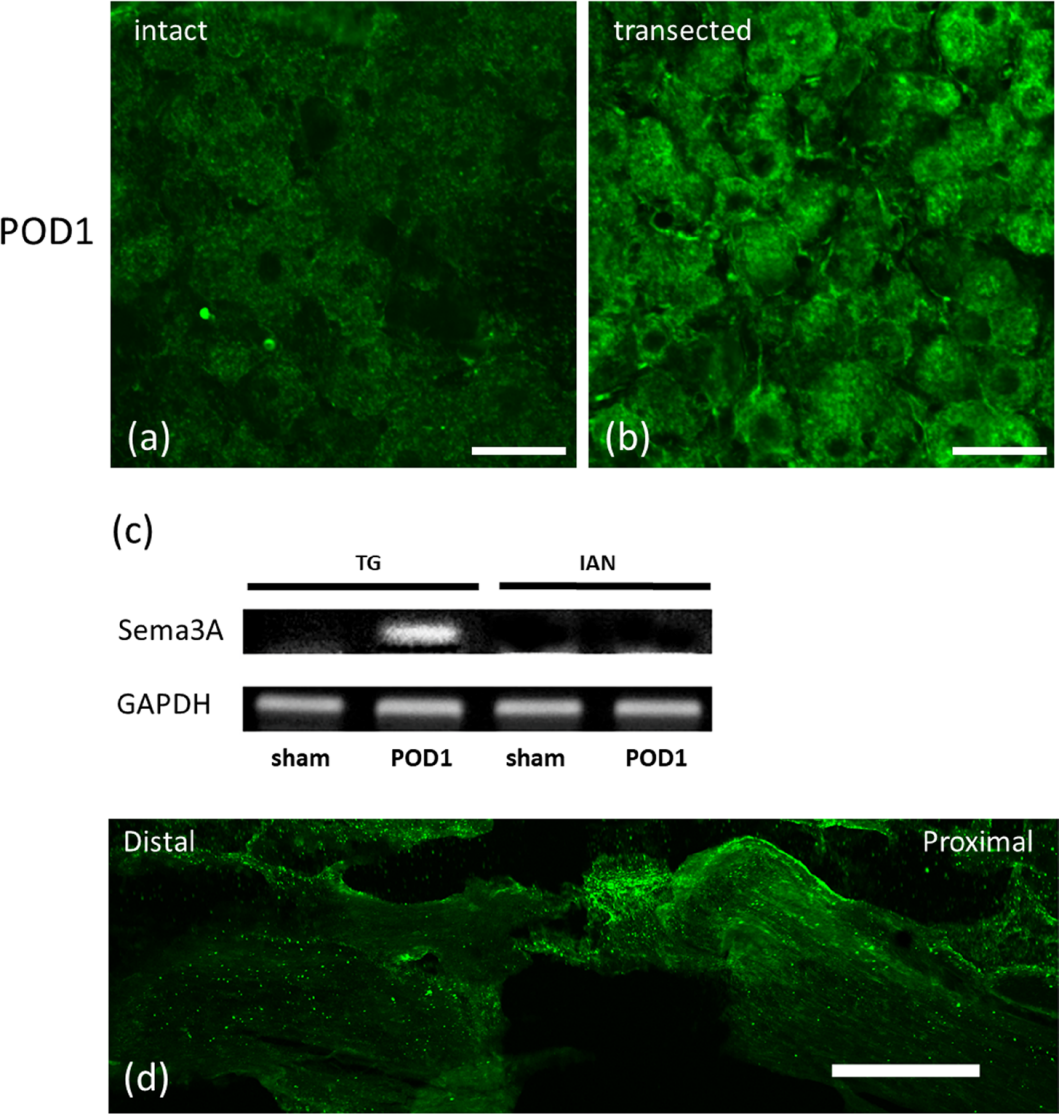
Immunohistochemical and RT-PCR analysis of Sema3A and neuropilin expression. Immunohistological observation of Sema3A expression in the trigeminal ganglion of intact (**a**) and transected IANs at postoperative (PO) day 1 (**b**). Sema3A immunoreactions are found in the cytoplasm of trigeminal neurons at PO day 1. (**c**) An single image of RT-PCR analysis showing expression of Sema3A in the trigeminal ganglion (TG) and inferior alveolar nerve (IAN) transection site in a sham animal and in an IAN transected animal at PO day 1. At PO day 1, an expression of Sema3A is observed in the trigeminal ganglion neurons. In contrast, there is not apparent expression of Sema3A in the region adjacent to the transection site of the IAN. Representative of RT-PCR analysis is shown (sham and PO day 1[POD1]). (**d**) Neuropilin expression in trigeminal ganglion of the transected IAN at PO day 1. Scale bars = 50 µm in (**a**,**b**) and 200 µm in (**d**).

Immunohistochemical survey was carried out for clarifying changes in immunoexpression pattern of Sema3A in the IAN and trigeminal ganglion after nerve injury. Protein gene product 9.5 (PGP9.5), a general neuronal marker, was used for the demonstration of nerve fibers during regeneration. We failed to find any Sema3A expression in the trigeminal ganglion or IAN in the absence of nerve transection (Figs 1a and 2a). At postoperative (PO) day 1, Sema3A expression was recognizable in the proximal stump of the IAN. This apparent immunoreaction was restricted to the proximal region of IAN, not in the distal region. Sema3A expression appeared weaker along the nerve fibers towards the center of the site and was completely absent from areas far from the transected lesion. At PO day 3, Sema3A immunoreactivity became weaker in the proximal stump (Fig. 1b,c), and was not found in the medial end of the distal stump. At PO day 7, immunohistochemistry for PGP9.5 demonstrated the connection of nerve bundles from the proximal site to the distal site, suggesting the occurrence of regeneration in the damaged IAN. However, Sema3A expression disappeared completely throughout the whole length of the IAN.

The trigeminal ganglion also had apparent expression of Sema3A in the cytoplasm at PO day 1 (Fig. 2b). In addition to protein expression *in vivo*, we examined gene expression of Sema3A in trigeminal ganglion. RT-PCR analysis at PO day 1 exhibited a band for Sema3A mRNA around 150 bp at the trigeminal ganglion, but not at the IAN (Fig. 2c). Amplified products for Sema3A mRNA were recognized in sham groups of neither trigeminal ganglion nor IAN. Immunoreactivity of neuropilin, a semaphorin 3 receptor, was also detectable at the end lesion of the proximal stump of the injured IAN at PO day 1 (Fig. 2d).

### Facilitatory effect of anti-Sema3A antibody administration on IAN regeneration

**Figure 3:**
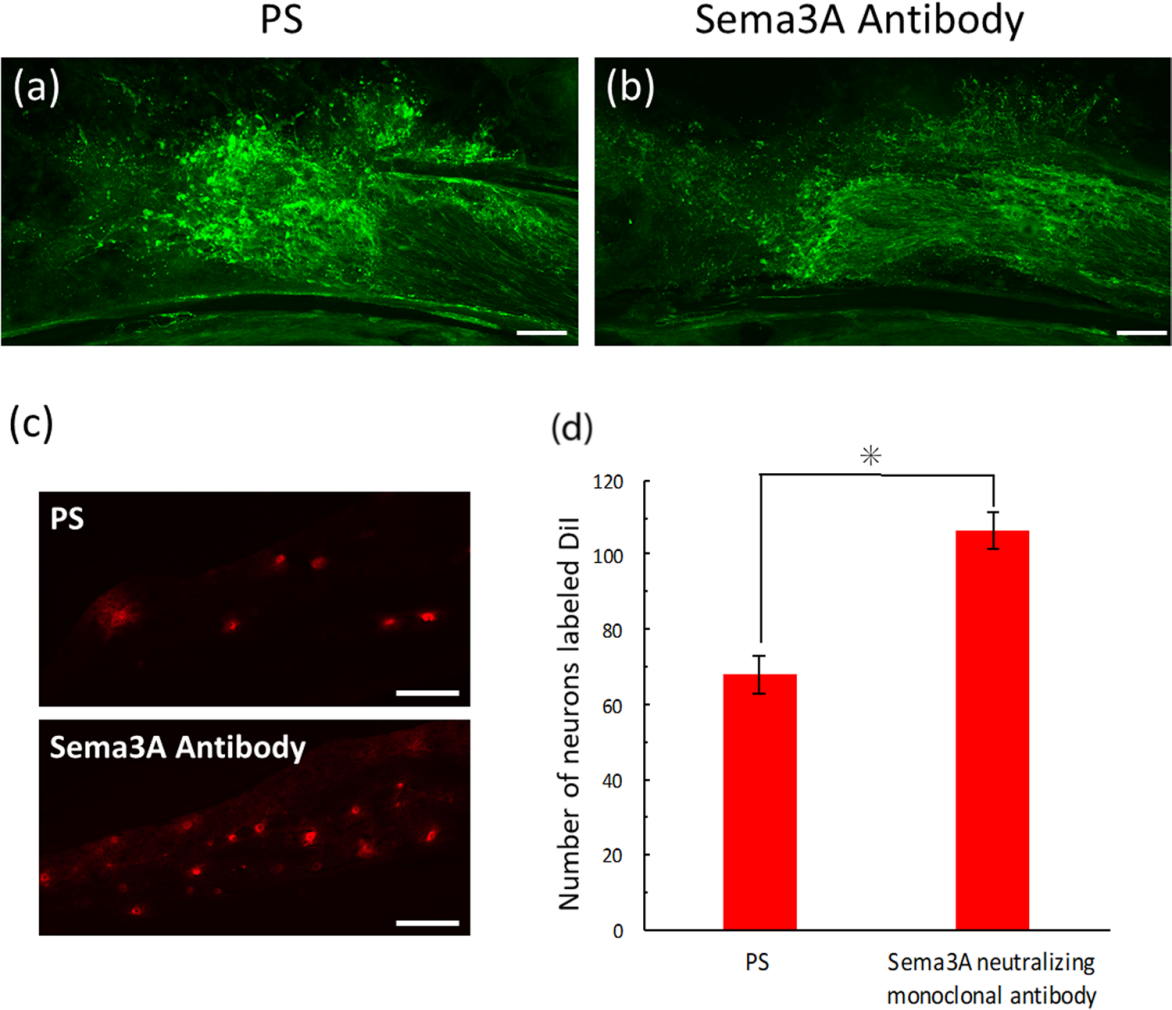
Effects on IAN regeneration with a local administration of anti-Sema3A antibody. (**a**,**b**) Nerve regeneration demonstrated with PGP9.5 immunohistochemistry at postoperative (PO) 3 days. (**a**) Nerve fibers extending in random direction from the lesion are observed in a vehicle control treated with physiological saline (PS). In contrast, administration of anti-Sema3A antibody eliminates the radial extension of injured axons, resulting in axonal regeneration (**b**). (**c**) A comparison of DiI-labeled neurons in the trigeminal ganglion with a local administration of PS and Sema3A antibody to the transected site of IAN. The graph (**d**) shows that the number of DiI-labeled neurons is greater in anti-Sema3A antibody-treated animals than in vehicle control animals with a significant difference (Student’s t-test, *p < 0.05). Scale bars = 100 µm (**a**–**c**).

Next, we investigated the effect of an antibody to Sema3A on the regeneration of IAN by immunohistochemistry to confirm the involvement of Sema3A in IAN regeneration. The anti-Sema3A antibody used in this study has been reported to neutralize the effects of Sema3A *in vitro*^11^. Nerve fibers extending in random directions from the lesion were observed in the vehicle control animals treated with physiological saline (PS) (Fig. 3a). In contrast, a local administration of an antibody to Sema3A in the transection site three times after nerve injury showed the projection of the transected nerve fibers from the proximal stump towards the distal stump, appearing as a reconnection of transected axons (Fig. 3b), indicating that antibody to Sema3A specifically inhibited the effect of Sema3A *in situ*.

In addition, we performed DiI retrograde tracing method for the demonstration of re-connection of the injured IAN by a local administration of DiI around mental foramen. DiI-labeled trigeminal ganglion neurons increased in number after an administration of the anti-Sema3A antibody as compared with the vehicle control group with PS-injection (Fig. 3c). This increase was confirmed to be statistically significant between PS- and Sema3A antibody-treated groups (Student’s t-test, p < 0.05) (Fig. 3c).

## Discussion

Previous reports have shown activation of Sema3A in response to peripheral nerve injury. Current immunostaining and RT-PCR analyses clearly demonstrated the expression of Sema3A in the ganglion as well as in the proximal stump of the transected IAN at PO day 1, indicating that this protein was delivered through the nerve fibers to the proximal stump distally. This was sensory in nature, suggesting the involvement of this molecule at an early stage of nerve regeneration rather than at later stages, as previously reported^12,13^.

### Sema3A expression after peripheral nerve injury

A previous study on human traumatic neuroma in the brachial nerve plexus suggested that fibroblasts secrete Sema3A into the epineurial space of peripheral nerve lesions or neuroma^7^. The present study was also able to detect Sema3A in the extra-fascicular space, which contains neither Schwann cells nor nerve fibers^7^. These findings led us to postulate that fibroblasts in the epineurium play a crucial role in neuroma formation.

Previous investigations have shown that time courses of Sema3A mRNA expression after peripheral nerve injury differed between motor and sensory neurons^13^. In an experimental crush model of motor nerves, Pasterkamp *et al*.^12^ reported the commencement of decreased Sema3 mRNA expression on PO day 4, followed by reappearance on PO day 7. In contrast, the central end of sensory nerves exhibited a gradual increase in Sema3A expression after injury^13^, consistent with the current finding of Sema3A expression at the central nerve end at PO day 3, but not at PO day 7. However, an experimental study of sciatic nerve transection demonstrated an induction of elevated Sema3A mRNA expression in dorsal root ganglia at PO day 3, but crush injury did not induce any change, suggesting that the type of injury is a factor in Sema3A expression. These expression patterns may be explained by the continuity of either nerve fibers or the perineurium, and by the different nature of nerve fibers as suggested by Lindholm^14^.

### Sema3A receptor expression after nerve transection

There have been controversies over the expression pattern of mRNA for neuropilin which can bind the semaphorin 3 receptor to regulate axonal guidance: why it was unchanged in dorsal root ganglion neurons^12^, but increased in dorsal horn nociceptive neurons^15^. A past quantitative RT-PCR analysis demonstrated the elevation of mRNA for neuropilin-1 and neuropilin-2 in the distal stump of the transected nerve fibers, indicating that neurons are not a target cell for Sema3A as shown by Scarlato *et al*.^16^. Generally, Sema3A is considered to bind neuropilin-1/plexin A on the surface of growth cones to form a complex with Axin-1, resulting in endocytosis of the Sema3A-neuropilin-plexin A complex. These complexes are retrogradely transported through nerve fibers, whereby they serve as repulsive axon guidance cues^17^. This idea is consistent with the present results indicating an emergence of neuropilin in the central site of the injured IAN. Taken together with previous findings, it appears that IAN nerve injury induces temporal elevation of Sema3A receptors to deliver Sema3A-neuropilin-plexin A complexes to trigeminal ganglion neurons projecting to the IAN.

### Physiological roles of Sema3A in peripheral nerve regeneration

Sema3A binds neuropilin-1 onto filopodial tips of growth cones with high affinity^18^. In this study, a local administration of anti-Sema3A antibody facilitated regeneration of transected nerve fibers and convergence of radiative sprouting from the injured nerve end of the IAN. This finding supports the idea of repulsive guidance by Sema3A on elongating regenerative axons.

It is interesting that the proximal stump expressed both Sema3A and neuropilin during the early stages after injury. It has been reported that the initial reaction to nerve injury in Wallerian degeneration occurs within 24 hours^19^, whereas the distal isolated portion of transected nerve fibers degenerates over the following several days after injury^20^. During this period, the neuronal cell bodies with nerve injury exhibit displacement of the nucleus to an eccentric position. One week after injury, macrophage recruitment commences to develop and activate the growth cone. Therefore, it is reasonable to consider that induction of Sema3A at an early stage is necessary to inhibit the initiation of regeneration because the growth cone activation is unprepared for the elongation of nerve fibers at this time. Thereafter, neurotrophin is produced locally in the lesion, whereby it may suppress the inhibitory effects of Sema3A on regeneration.

The IAN is a branch of the trigeminal nerve containing sensory neurons. Differences in nature between sensory and motor nerves may explain the varying expression sites of Sema3A and neuropilin: that is, at the proximal site of transected IAN nerves (present study) versus at the distal site of injured motor nerves^13^. The involvement of Sema3A-neuropilin signaling in the regeneration process at the proximal stump of transected sensory nerves may indicate that nerve injury actively inhibits the neuronal cytoskeletal re-organization that facilitates fasciculation^7^ and axoplasmic transport^21,22^. In addition, Sema3A has been reported to induce axonal facilitation in adult dorsal root ganglion neurons in which Sema3A collapses NGF-facilitated axonal branching in embryonic neurons^23^. Although it seems likely that this interaction induces neuroma formation after nerve injury, the physiological implications remain unclear. Further investigation will have to clarify the coexistence of Sema3A-mediated inhibition and neurotrophin-mediated facilitation of nerve regeneration.

## Conclusion

This *in vivo* study demonstrated that transection of IAN induced simultaneous expression of Sema3A and neuropilin on PO day 1 at the central side of the injured IAN. A local administration of a Sema3A-antibody to the injury lesion facilitated regeneration and caused an increase in number of DiI-labeled neurons in the trigeminal ganglion. Therefore, Sema3A-neuropilin signaling is possibly activated at the early stage of peripheral nerve regeneration after IAN transection, leading to inhibition of injured nerve regeneration and affecting neuroma formation.

## Materials and Methods

### Animals

All the animal experiments were conducted in compliance with the protocol which was reviewed by the Institutional Animal Care and Use Committee and approved by the President of Niigata University (Approval Number: 27 Niigata Univ., Res 227-4). All methods were performed in accordance with relevant guidelines and regulations. Animals were housed under controlled temperature (25 °C) and humidity (approximately 40%) with a 12-h light/dark cycle and free access to food and water.

### General procedures

Forty-three male C57BL/6J mice (7–8 weeks, weighing 22–26 g) (Charles River Laboratories Japan, Inc., Yokohama, Japan) were used in this experimental study. Transection of the IAN was performed according to previously described methods^10^. Briefly, under anesthesia with inhalation of sevoflurane and an intraperitoneal injection of 4% chloral hydrate (2.5 g/kg), the IAN from the mandibular canal was transected at one side, the cut ends of the nerve were returned into the canal, and the wound was then sutured (n = 12). Four mice who received an operation for IAN exposure without nerve resection served as a control group. No postoperative treatment, such as administration of antibiotics or analgesics, was given to these mice because clean, limited invasion surgeries induced no inflammation symptoms and recovery well from the operation was uneventful in all animals. Animals with nerve resection were sacrificed at PO days 1, 3 and 7 (4 animals in each PO day), and four animals in the control group at PO day 7. They were transcardially perfused with 4% paraformaldehyde in 0.1 M phosphate buffered-saline under deep anesthesia using sevoflurane. Following decalcification with 10% ethylene diamine tetra-acetic acid (EDTA) solution for 3 weeks, sagittal cryostat sections of mandibles including the IAN bundle were prepared at a thickness of either 20 μm or 70 μm in a cryostat.

Sections were processed for immunofluorescent method according to our previously published protocol^10^. Briefly, sections were incubated with primary antibodies shown in Table 1 at 4 °C overnight, and then treated with an appropriate secondary antibody (Table 1) at room temperature for 1 hour. For making faint reaction visible by immunoenhancement, tyramide signal amplification was performed for Sema3A antibody detection according to the manufacturer’s instructions (Perkin Elmer Life Science, Waltham, MA). Fluorescent images were captured with either a fluorescent microscope (Axioimager M1; Carl Zeiss, Oberkochen, Germany) or confocal laser-scanning microscope (LSM 700; Carl Zeiss). We did not perform any image processing operation including adjustment of contrast or brightness throughout this study.

**Table 1:** List of antibodies used in this study.

Antibody	Dilution	Company	Catalogue No.
Sema3A	1:500	Abcam (Cambrige, UK)	ab23393
Neuropilin	1:100	Abcam (Cambrige, UK)	ab81321
PGP9.5	1:1,000	Ultraclone (Wellow, UK)	RA-95101
FITC anti-rabbit	1/300	Vector (California US)	FI-1000
Texas Red anti-rabbit	1/300	Vector (California US)	TI-1000

### Local administration of anti-Sema3A antagonist

Following nerve transection, 10 μL (50 μg) of anti-Sema3A monoclonal antibody which was courtesy with Chiome Bioscience Inc., Tokyo, Japan was locally administered by a Hamilton Syringe to the transected area three times: immediately after surgery and at PO day 1 and PO day 2 under anesthesia by sevoflurane inhalation (n = 4). This anti-Sema3A antibody has proven effective in neutralizing the effects of Sema3A *in vitro*^11^. As a vehicle control (n = 3), 10 μL of PS was applied in the same manner.

### DiI retrograde tracing in the trigeminal ganglion

**Figure 4:**
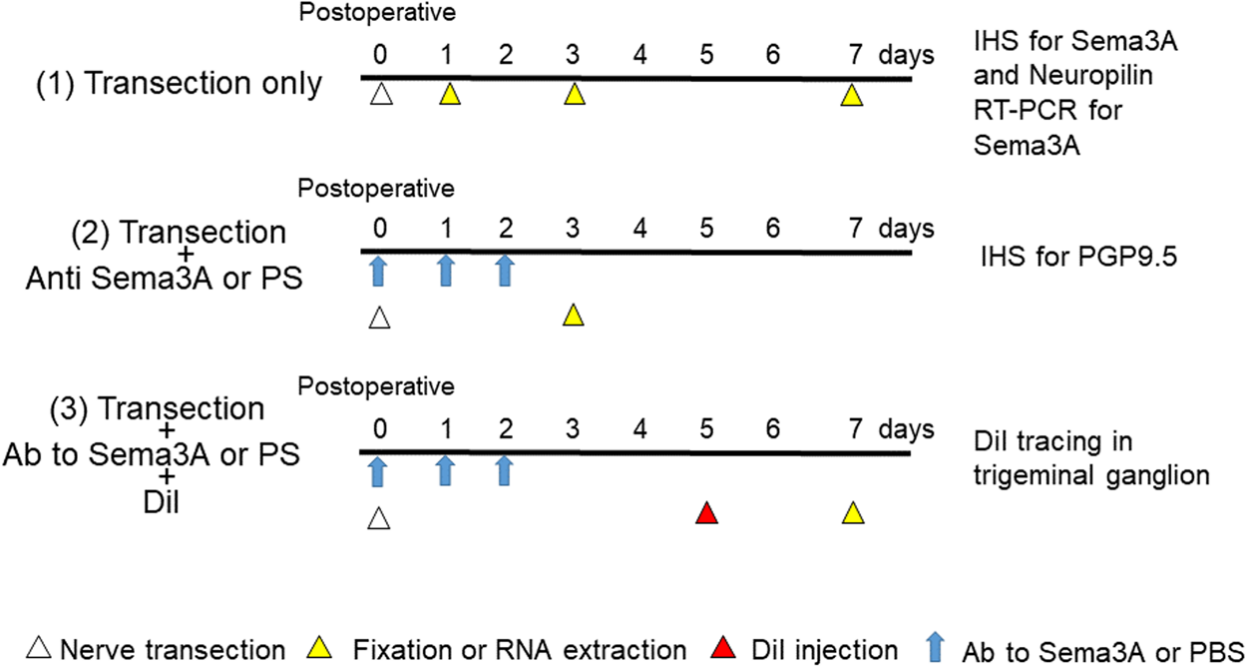
Diagram of this experimental design. The experiments in this study are largely divided into three categories (1–3). After nerve injury, individual experimental procedure was carried out at appointment time. Ab: antibody, IHS: immunohistochemistry, PS: physiological saline.

To investigate effects of Sema3A on nerve regeneration, an additional eight mice were used to identify regenerated nerve fibers in the trigeminal ganglion by a 1,1′-dioctadecyl-3,3,3′,3′-tetramethylindocarbocyanine perchlorate (DiI) labeling technique. Under anesthesia with inhalation of a high concentration of sevoflurane, a subcutaneous injection of 3 μL of DiI (Invitrogen, Waltham, MA) was carried out around the mental foramen using a Hamilton syringe at PO day 3 (see, Yamada *et al*.^10^). Vehicle control used PS injection instead of DiI injection. These mice were sacrificed 2 days after DiI injection to examine trigeminal ganglion neurons that had been labeled by peripheral dye injection. DiI-labeled neurons in each animal were counted under a fluorescence microscope (three sections per animal). Numbers of DiI-labeled trigeminal ganglion neurons were compared by Student’s t-test between PS- and anti-Sema3A antibody-treated animals (n = 4 in each group). Time-course of this experimental design is summarized in Fig. 4.

### Reverse-transcription polymerase chain reaction (RT-PCR) for Sema3A

Samples of nerve lesions for regenerated IAN were removed from the mandibular nerve in a 10-mm length containing the cut lesion. As isogen solution (Nippon Gene Co., Ltd., Tokyo, Japan) was used to extract total RNA from the IAN and trigeminal ganglion with or without IAN transection (n = 6 in each group). RT-PCR was performed using an Access RT-PCR kit (Promega Corporation, Madison, WI) according to the manufacturer’s instructions. The PCR reaction was carried out in the presence of gene specific Sema3A (forward 5ʹ-CAGCCATGTACAACCCAGTG-3ʹ and reverse 5ʹ-ACGGTTCCAACATCTGTTCC-3ʹ [size: 150 bp] and GAPDH (forward 5′‐GGCACAGTCAAGGCTGAGAATG‐3′and reverse 5′-ATGGTGGTGAAGACGCCAGTA‐3′) primers. Isolated RNA without reverse transcription obtained from the samples was used as a negative control. Amplified products were separated by electrophoresis in 2.5% agarose gels, and the gels stained with ethidium bromide were examined by ultraviolet transillumination (Funakoshi, Tokyo, Japan). The photograph was taken as a single image with a digital camera.

### Statistical analysis

Numbers of DiI-labeled trigeminal ganglion neurons between PS- and anti-Sema3A antibody-treated animals was compared by Excel for Mac2011 (Microsoft, Redmond, WA). A p-value of less than 0.05 was considered significant. Normality was analyzed by the Shapiro-Wilk normality test for the control and antibody-treated animals.
